# Exposing the Airway Surface to the Neonicotinoid Clothianidin Alters the Electrophysiological Properties of Human Airway Epithelia

**DOI:** 10.1021/acsomega.5c09156

**Published:** 2026-01-15

**Authors:** Darrin A. Thompson, Vivek Kumar Srivastava, Lucy A. Siwicki, Andrea Adamcakova-Dodd, Emma M. Stapleton, Ian M. Thornell

**Affiliations:** † Center for Health Effects of Environmental Contamination, University of Iowa, Iowa City, Iowa 52242, United States; ‡ Department of Occupational and Environmental Health, College of Public Health, 145 N Riverside Dr, University of Iowa, Iowa City, Iowa 52242, United States; § Department of Internal Medicine and Pappajohn Biomedical Institute Roy J. and Lucille A. Carver College of Medicine, University of Iowa, Iowa City, Iowa 52242, United States

## Abstract

The neonicotinoid clothianidin is used indoors and outdoors as an insecticide and extensively as a seed coating in agriculture where working solutions are prepared at 4000 ppm. Health effects of clothianidin are often studied under the assumption that ingestion is the exposure route, yet inhalation exposures are likely, given that clothianidin is sprayed and dust is resuspended. We studied the effect of airway exposure to clothianidin using human donor epithelia. Acute clothianidin doses greater than 50 ppm applied to the apical (airway-facing) surface resulted in decreased ion transport properties, specifically, decreased activity of the surface sodium channel, ENaC. During a 6 h 500 ppm clothianidin exposure, the permeability of airway epithelia to clothianidin rose from ∼4.2 × 10^–6^ to ∼13.5 × 10^–6^ cm·s^–1^ without an increase in cell death, indicating a loss of barrier integrity. Respiratory precautions should be considered for those in proximity to aerosol-generating clothianidin application.

## Graphical abstract

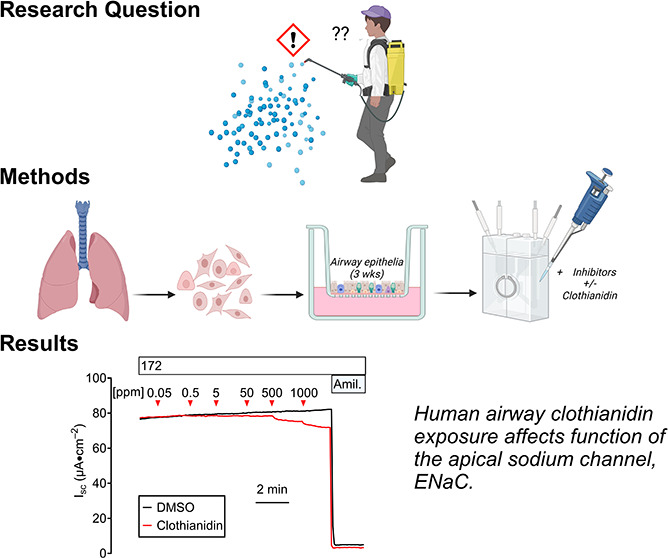

## Introduction

In the United States, roughly 1 billion pounds of conventional pesticides are applied annually for agricultural and pest-related concerns. Neonicotinoids are a major component of pesticides, as they target insects with higher specificity than organophosphates. Neonicotinoids typically inhibit insect nicotinic acetylcholine receptors with 100-fold greater affinity compared to those of mammals.^1^ Several studies report that neonicotinoids and their metabolites have biological activity in animal models (e.g., mice).^2^ One of these neonicotinoids, clothianidin, is commonly applied to seeds, including corn kernels. It is released at a rate of approximately 26.5 g/active ingredient/acre in heavy agricultural areas, like Iowa.^3^ Commercial and residential solutions for spraying are prepared at 4000 ppm clothianidin. Little is known about the biological effects of these compounds on human cells, especially on the surface of the airways. Here, we tested whether inhalational exposure to clothianidin generates a biological response in primary human airway epithelia cultured at the air–liquid interface.

## Methods

### Human Airway Epithelia

Differentiated primary human airway epithelia were obtained from the University of Iowa cell culture core according to their published protocol and approved by the University of Iowa Institutional Review Board.^4^ Airway epithelia were cultured >21 days at the air–liquid interface in basolateral media containing 1:1 DMEM/F-12 (Gibco) supplemented with 1% penicillin/streptomycin and 2% serum substitute UltraSer G (Pall BioSeptra). After 21 days, cultured epithelia form a pseudostratified monolayer containing all epithelial cells found in human airways.^4,5^

### Transepithelial Voltage Clamp Technique

To test the acute effect of clothianidin, we measured the short-circuit current (*I*_sc_) and transepithelial conductance (*G*_t_), which measure the ion transport properties of an epithelium. Epithelia were mounted into Ussing Chambers (P2300, Physiologic Instruments) and evaluated using the transepithelial voltage clamp technique. Each hemichamber was filled with 5 mL of Krebs solution (in mM: 135 NaCl, 2.4 K_2_HPO_4_, 0.6 KH_2_PO_4_, 1.2 CaCl_2_, 1.2 MgCl_2_, 5 HEPES, 5 glucose; salts obtained from Sigma-Aldrich) and continuously gassed through the chamber inlets with air. The electrodes were bathed in 3 M KCl and connected to the chambers using agar bridges (3% agar in 3 M KCl); the electrode wire was connected to an amplifier (VCC-MC8, Physiologic Instruments). The voltage clamp was controlled through Acquire and Analyze software (v2.3, Physiologic Instruments). The transepithelial voltage was held at 0 mV, and the short-circuit current (*I*_sc_) was measured. An intermittent ±5 mV pulse of 200 ms was applied to obtain the transepithelial conductance (*G*_t_). All compounds used in the assays were purchased from Sigma-Aldrich. Data were exported into Microsoft Excel and then analyzed using a custom MATLAB (v2023a) script developed by Ian Thornell (available upon request).

### Dose and Time Points

Fresh clothianidin was initially prepared as a 500,000 ppm DMSO stock solution. Using the 500,000 ppm clothianidin stock solution, each dose was prepared as 1000x stock to ensure each contributed 0.1% DMSO to the experimental solution. To test the acute effects of clothianidin, a dose response from 0.05 to 1000 ppm (final concentration) was applied to the solution bathing the apical (airway-facing) surface of an airway epithelium. To test the chronic effect of clothianidin, the apical surface of cultured human airway epithelia was exposed to 2 μL of 500 ppm clothianidin in phosphate-buffered saline containing divalent cations for 2 and 6 h, then the short-circuit current (*I*_sc_) and transepithelial conductance (*G*_t_) were measured in the absence of clothianidin. Donor-matched DMSO-treated epithelia were used as controls for each experiment.

### Clothianidin Permeability

To test the rate at which airway epithelia absorb clothianidin, epithelia were mounted into Ussing chambers and bathed in a Krebs solution. Clothianidin (500 ppm) was applied to the apical chamber, and 100 μL of apical and basolateral solution was removed after ∼15 s, 2 min, 20 min, 1, 2, and 6 h. The absorbance values for 264 nm light were obtained from samples using a SpectraMax i3× (Molecular Devices), after correction for path length and background absorbance using donor-matched conditioned Krebs solution. An extinction coefficient of 0.088 M^–1^·cm^–1^ for a 264 nm light excitation was used to estimate the apical and basolateral [clothianidin].^6^

The permeability of airway epithelia to clothianidin *P*_clothianidin_ was estimated by Fick’s law of diffusion^7^

$$P=\frac{J}{C^{a}-C^{b}}$$

where *P* is the permeability of the airway epithelia (cm·sec^–1^), *J* is the clothianidin flux (mol·s^–1^·cm^–2^) based on the appearance of clothianidin in the basolateral chamber, *C* ^*a*^ is the apical clothianidin concentration (mol·cm^–3^), and *C* ^*b*^ is the basolateral clothianidin concentration (mol·cm^–3^).

### Cell Viability Assays

Basolateral media from chronic exposure (see the Dose and Time Points section) was collected from each epithelia and used to wash the apical surface. The wash was assayed for lactate dehydrogenase (LDH) activity, while the cells were processed for live–dead staining. LDH activity of the wash was tested using a CytoTox-ONE Homogeneous Membrane Integrity Assay kit (Promega), per manufacturer’s instructions. To calculate percentage cytotoxicity, culture medium background values were subtracted from all the experimental wells and reported as a percentage of maximum LDH release control (0.2% Triton X-100). For flow cytometry, cells were further washed twice with phosphate-buffered saline (PBS). Cells were stained at room temperature with a LIVE/DEAD Fixable Near-IR Dead Cell Stain (Invitrogen) for 10 min. After staining, cells were washed twice with PBS and dissociated using Accutase cell dissociation solution (Innovative Cell Technologies, Inc.) for 30 min at 37 °C. Single-cell suspensions were fixed and permeabilized with an eBioscience FOXP3/Transcription factor staining buffer set (Invitrogen) at 4 °C for 30 min. Cells were then resuspended in 200 μL of flow buffer, and data were acquired using a flow cytometer (Attune NxT flow cytometer, Invitrogen, USA).

### Analysis

Each *n* value in a single treatment group represents a genetically distinct human donor epithelium. Statistics were performed using GraphPad Prism v10.4.1. Shapiro-Wilk testing revealed that all data are normally distributed; therefore, paired donor data were analyzed using paired *t* test or ANOVA with Bonferroni correction for multiple comparisons, and *P* ≤ 0.05 was regarded as significant. All error values represent one standard deviation from the mean.

## Results and Discussion

### Acute Effects of Clothianidin Airway Surface Exposure

Ion transport across the airway epithelium serves many purposes, including maintenance of a thin coating of airway surface liquid to protect the host from inhaled exposures.^8^ The ion transport properties of the epithelium were assessed by measurement of the short-circuit current (*I*_sc_) and transepithelial conductance (*G*_t_) (experimental schematic, Figure 1A). Acute effects of clothianidin exposure to the airway surface by addition of clothianidin to the apical solution (airway surface bathing) were assessed by dose–response (∼2 min per dose); first, its effects on the epithelial Na^+^ channel (ENaC) were tested in the presence of the CFTR (cystic fibrosis transmembrane conductance regulator) inhibitor CFTR_inh_-172 (Figure 1B–D). Then, the remaining short-circuit current was blocked by the ENaC inhibitor amiloride. Donor-paired controls were treated with DMSO, then amiloride. Clothianidin modestly decreased amiloride-sensitive *I*_sc_ and *G*_t_ at doses >50 ppm, indicating an effect on the epithelial Na^+^ channel (ENaC). We did not observe an acute effect of clothianidin on CFTR when epithelia were studied in the presence of amiloride (Figure 1E–G).

**Figure 1:**
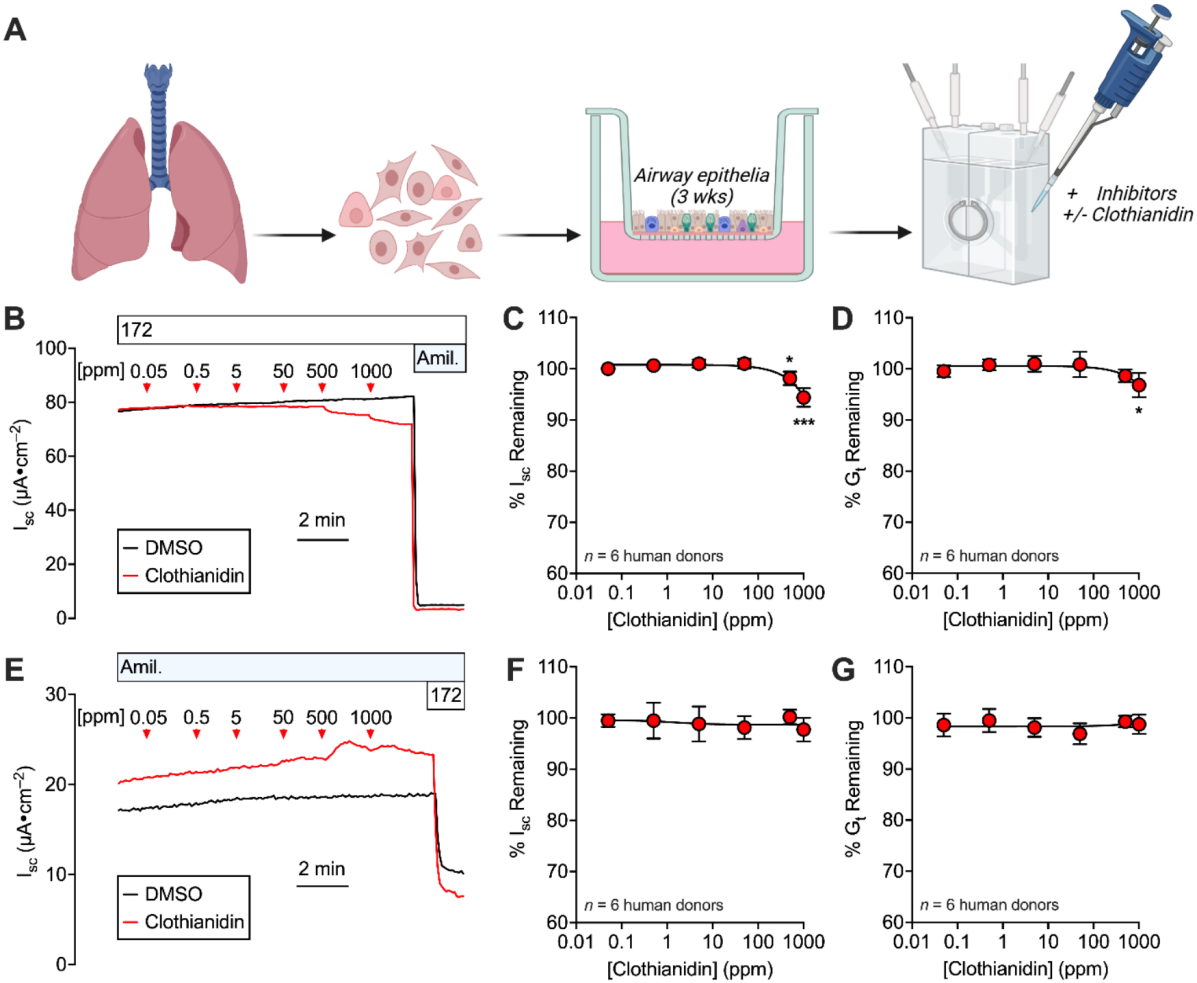
Acute clothianidin exposure to the airway surface affects amiloride-sensitive *I*_sc_ and *G*_t_. (A). Approach taken to study the acute effect of clothianidin on airway epithelia. (B). Example short-circuit current recording assessing the effect of clothianidin on ENaC-mediated electrolyte transport. Graphics created with BioRender.com. (C, D). Clothianidin dose–response for ENaC-mediated *I*_sc_ and *G*_t_. (E). Example short-circuit current recording assessing the effect of clothianidin on CFTR-mediated electrolyte transport. (F, G). Clothianidin dose–response for CFTR-mediated *I*_sc_ and *G*_t_.

### Acute Effects of Carbachol Airway Surface Exposure

Clothianidin is known to act through nicotinic acetylcholine receptors.^2^ Adding the acetylcholine analogue carbachol to the apical solution did not alter *I*_sc_ or *G*_t_, indicating that clothianidin did not activate a surface acetylcholine receptor in our model (Figure 2). The finding is similar to that reported by Joo et al., who found that carbachol alters electrolyte transport in airway epithelial cells only when applied to the basolateral (blood-facing), but not apical, solution.^9^ Additionally, the data indicate that carbachol did not diffuse across the epithelia to activate basolateral receptors.

**Figure 2:**
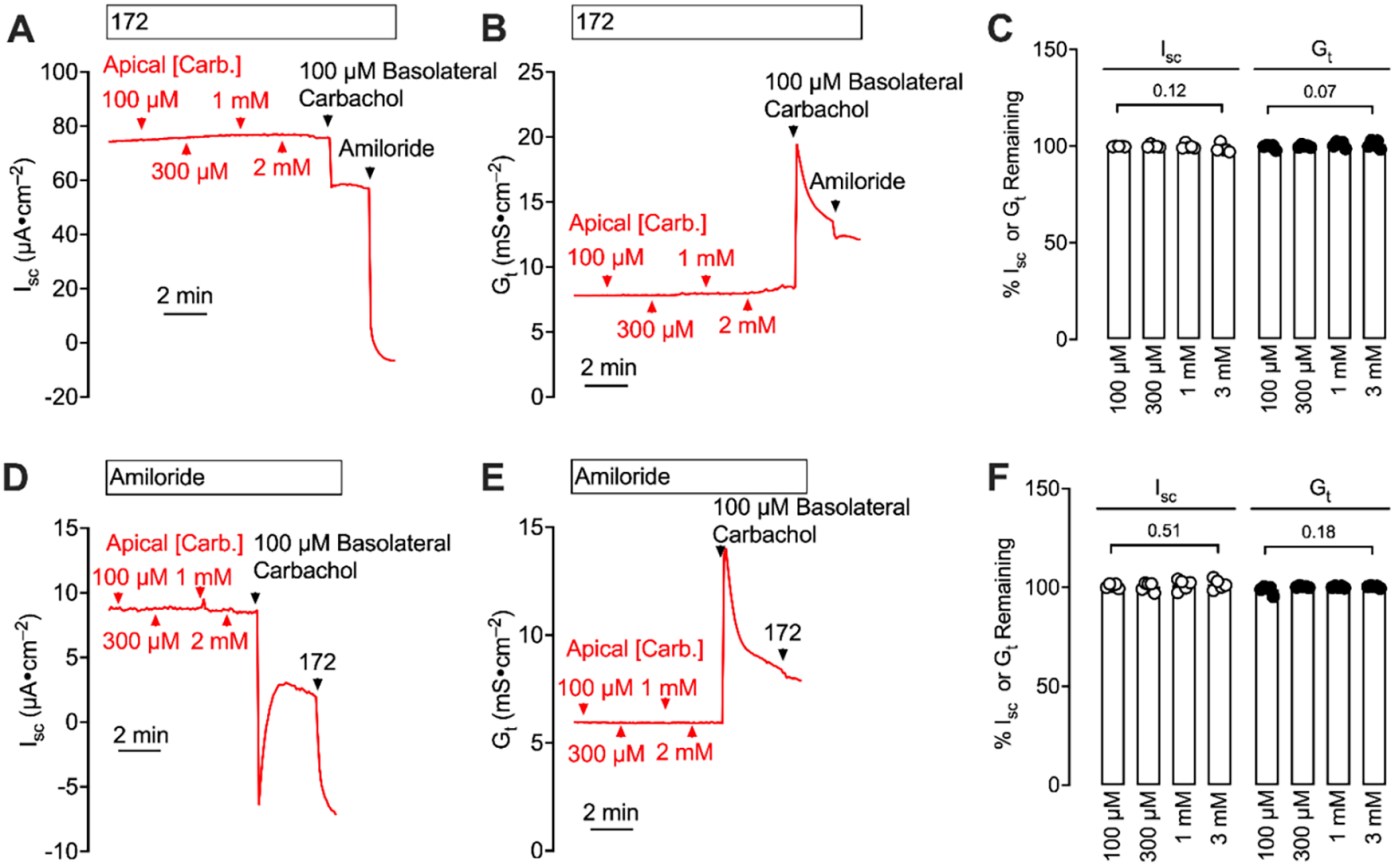
Acute carbachol exposure to the airway surface does not alter *I*_sc_ and *G*_t_. (A) Example short-circuit current recording assessing the effect of apical carbachol on ENaC-mediated electrolyte transport. Basolateral carbachol (100 μM) was added as a control. (B) Conductance recording corresponding to (A). (C) Apical carbachol dose–response for ENaC-mediated *I*_sc_ and *G*_t_. (D) Example short-circuit current recording assessing the effect of apical carbachol on CFTR-mediated electrolyte transport. Basolateral carbachol (100 μM) was added as a control. (E) Conductance recording corresponding to (D). (F) Apical carbachol dose–response for CFTR-mediated *I*_sc_ and *G*_t_.

Because airways may have different permeabilities to clothianidin and carbachol, we measured clothianidin in the apical and basolateral solutions after a 500 ppm exposure (Figure 3A). The slower accumulation of clothianidin in the basolateral chamber compared to the depletion of clothianidin in the apical chamber suggests that some clothianidin interacted with the chamber, complexed in the apical solution altering its spectra, and/or accumulated inside the epithelia. For each time point, we used the rates of clothianidin appearance in the basolateral solution and concentration gradients to calculate the permeability of human airway epithelia to clothianidin. Initially, the permeability was 4.2 ± 0.08 × 10^–6^ cm·s^–1^ then approached 13.5 ± 3.8 × 10^–6^ cm·s^–1^ after 6 h (Figure 3B). As a reference, the permeability of the pathways between cells for monovalent ions is 5 × 10^–6^ to 7 × 10^–6^ cm·s^–1^.^10^ Based on these values, there was nominal accumulation of clothianidin in the basolateral chamber in experiments performed for Figure 1.

**Figure 3:**
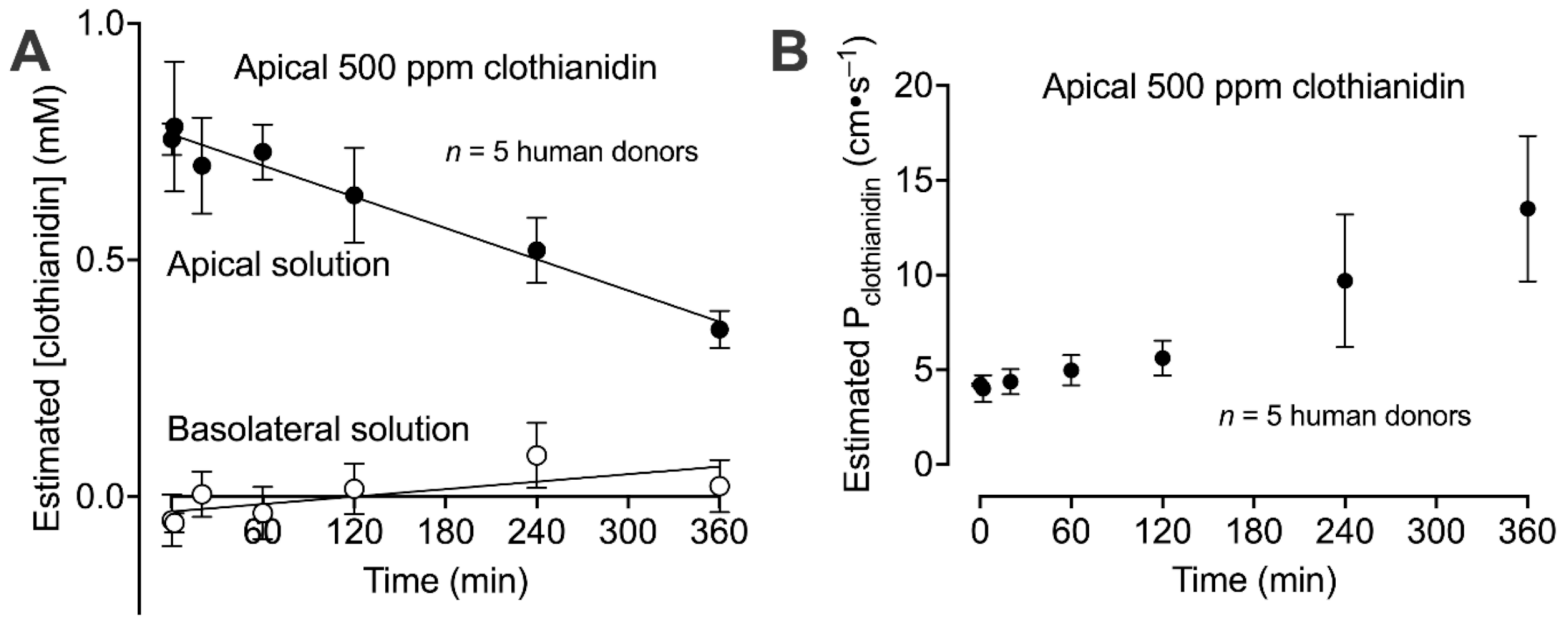
Increase in apparent clothianidin permeability over time. (A) Clothianidin concentration in apical and basolateral solution bathing airway epithelia determined by absorption of 264 nm light during a 500 ppm apical clothianidin exposure. (B) Rate of clothianidin appearance in the basolateral solution and its concentration gradient for each time point used to estimate the permeability of airway epithelia to clothianidin.

### Chronic Effects of Clothianidin

The apical surface of cultured human airway epithelia was pretreated with 500 ppm of clothianidin for 2 and 6 h, then short-circuit current (*I*_sc_) and transepithelial conductance (*G*_t_) were measured in the absence of clothianidin (Figure 4A). We sequentially added the ENaC inhibitor amiloride, then forskolin to increase CFTR activity, and then CFTR inhibitor CFTR_inh_-172 (Figure 4B–E). Two-hour clothianidin pretreatment did not alter *I*_sc_ values, suggesting acute effects of clothianidin on amiloride-sensitive *I*_sc_ are reversible (Figure 4B). However, the *G*_t_ values increased throughout the assay (Figure 4C). Elevated *G*_t_ values without altered *I*_sc_ values suggest a breakdown of the epithelial tight junctions. Interestingly, the effect on *G*_t_ reversed at the 6 h time point (Figure 4D,E). Chronic clothianidin treatment did not increase epithelial lactate dehydrogenase release (Figure 5A) nor affect live/dead stain permeability (Figure 5B). Although it remains unclear why *G*_t_ reversed while clothianidin permeability increased at 6 h (Figure 3), it may be that the apical clothianidin concentration is driving the effect on *G*_t_.

**Figure 4:**
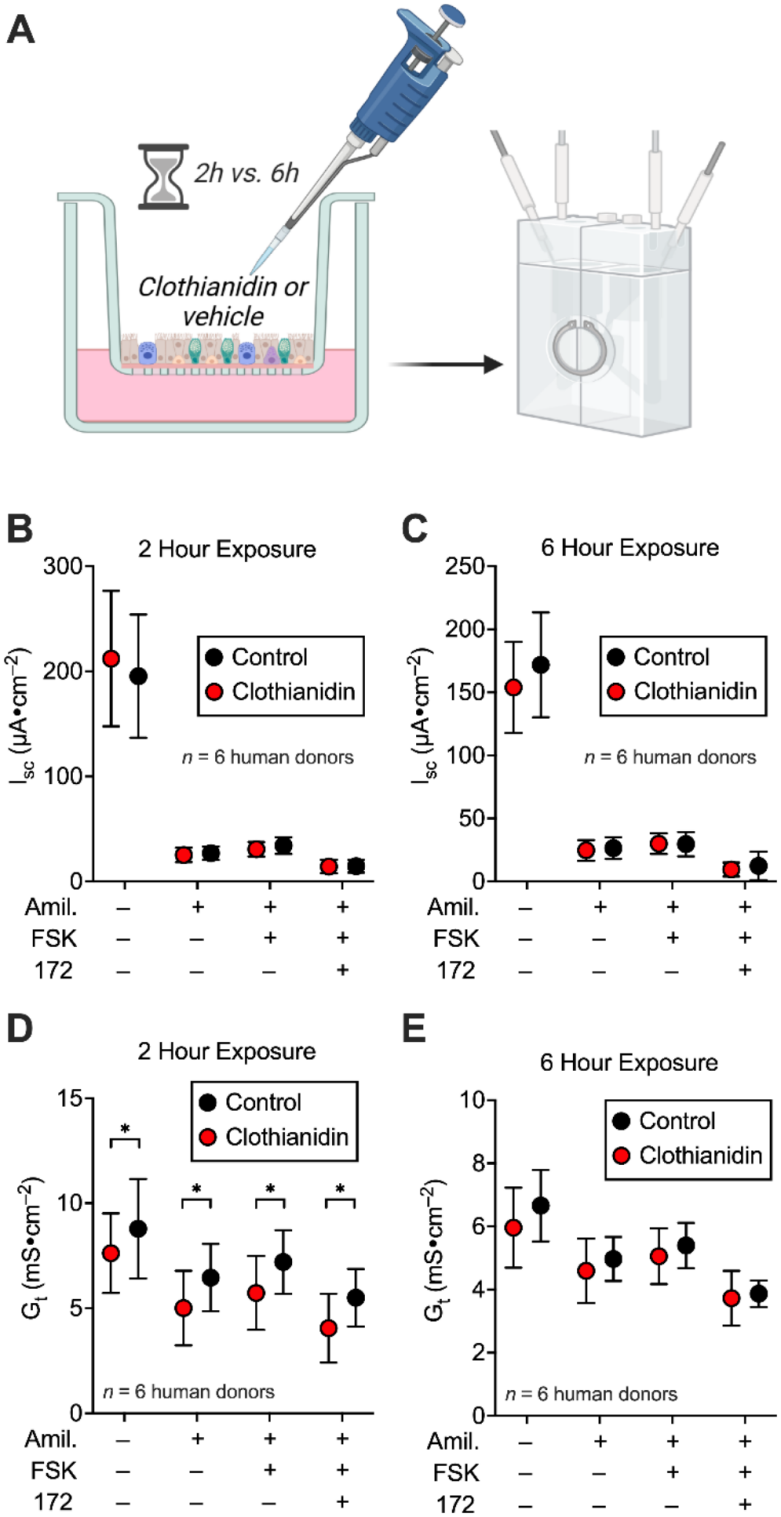
Prolonged clothianidin exposure to the airway surface affects *G*_t_. (A) Approach taken to study the effect of 500 ppm clothianidin on airway epithelia. Graphics created with BioRender.com. (B) *I*_sc_ data for airway epithelia exposed to clothianidin or vehicle for 2 h. (C) *I*_sc_ data for airway exposed to clothianidin or vehicle for 6 h. (D) *G*_t_ data for airway exposed to clothianidin or vehicle for 6 h. (E) *G*_t_ data for airway exposed to clothianidin or vehicle for 6 h.

**Figure 5:**
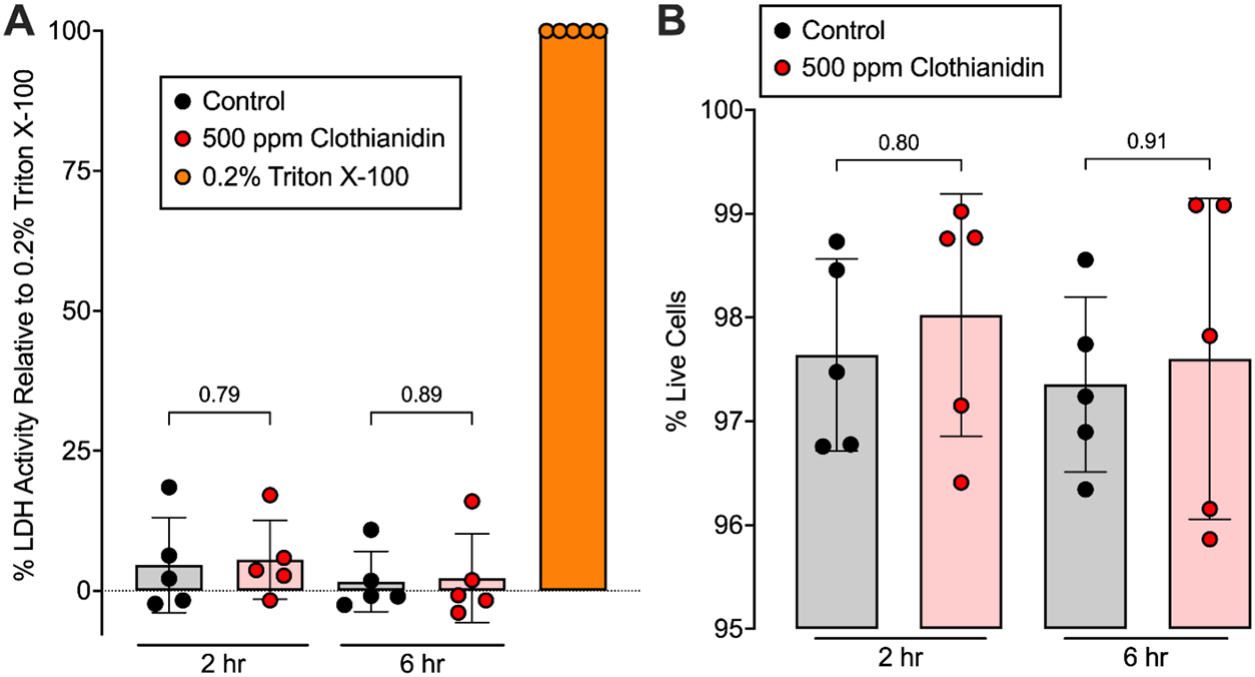
Prolonged 500 ppm clothianidin exposure does not kill airway epithelial cells. (A) Extracellular activity of intracellular enzyme lactate dehydrogenase in extracellular wash of epithelia as an indicator of cell lysis with Triton X-100 treatment as a positive control. (B) Live–dead stain of the same cells assayed by flow cytometry.

Our preparation lacks immune cells and nerves; therefore, our data indicate that clothianidin has biological activity at minimum on human airway epithelia, the first line of defense against inhaled pollutants. These findings contribute to a growing body of evidence that neonicotinoids, although designed for insect specificity, may exert unintended effects on human tissues. Rasmussen et al. reported that 5-day clothianidin exposure to the basolateral surface of airway epithelia decreased ENaC and CFTR currents via nicotinic receptors.^11^ Our study differs in several ways. We applied clothianidin to the airway surface to model an inhalation exposure. Our treatments were designed to model occupational or residential exposures that would represent a single workday. Our finding that the clothianidin permeability of airway epithelia increased at 6 h suggests that basolateral nicotinic receptors could play a role during a chronic exposure. However, our dose–response data for 2 min exposures more likely occurs through an apical or cellular mechanism. Targeting this mechanism could provide additional protection for those working with clothianidin.

Our data suggest that clothianidin acts on other targets in airway epithelia in addition to basolateral nicotinic receptors. For example, nonspecific receptors that sense a wide spectrum of chemical groups may be involved (e.g., aryl hydrocarbon and toll-like receptors).^12^ Clothianidin is known to contain *N*-nitroguanidine and thiazole functional groups; however, non-nicotinic airway receptors for these moieties remain unidentified. An additional possible mechanism could be driven by cytosolic clothianidin, which we were unable to measure. Mechanisms need not be mutually exclusive.

Given that we observed airway effects at concentrations beginning near 50 ppm and considering the epithelium’s high liquid absorption rate,^13^ respiratory precautions should be taken when clothianidin is sprayed. Working insecticide solutions contain up to 4000 ppm clothianidin for both residential and agricultural spraying.^2^ To date, there are no reports of direct measurements of neonicotinoids in airway surface liquid that could provide useful exposure data. This is needed because airways absorb liquid; thus, ambient measurements may not reflect airway surface liquid concentrations. In such studies, obtaining peak exposures during application events (or seasons) would be important to assess acute risks and relevant exposures.

From a public health perspective, these results underscore the importance of expanding toxicological screening to include airway models, particularly for compounds with an inhalation exposure potential. Such models may help inform future pesticide risk assessment frameworks, including the development of occupational exposure limits and inhalation safety guidelines. Based on our findings, inhalational exposure protection precautions should be taken when clothianidin is sprayed.
